# Cognitive Reflection, Decision Biases, and Response Times

**DOI:** 10.3389/fpsyg.2016.01402

**Published:** 2016-09-22

**Authors:** Carlos Alós-Ferrer, Michele Garagnani, Sabine Hügelschäfer

**Affiliations:** Department of Economics, University of CologneCologne, Germany

**Keywords:** cognitive reflection, decision biases, response times, decision making, Bayesian updating, multiple processes

## Abstract

We present novel evidence on response times and personality traits in standard questions from the decision-making literature where responses are relatively slow (medians around half a minute or above). To this end, we measured response times in a number of incentivized, framed items (decisions from description) including the Cognitive Reflection Test, two additional questions following the same logic, and a number of classic questions used to study decision biases in probability judgments (base-rate neglect, the conjunction fallacy, and the ratio bias). All questions create a conflict between an intuitive process and more deliberative thinking. For each item, we then created a non-conflict version by either making the intuitive impulse correct (resulting in an alignment question), shutting it down (creating a neutral question), or making it dominant (creating a heuristic question). For CRT questions, the differences in response times are as predicted by dual-process theories, with alignment and heuristic variants leading to faster responses and neutral questions to slower responses than the original, conflict questions. For decision biases (where responses are slower), evidence is mixed. To explore the possible influence of personality factors on both choices and response times, we used standard personality scales including the Rational-Experiential Inventory and the Big Five, and used them as controls in regression analysis.

## 1. Introduction

Human beings attempt to behave rationally, but they often struggle as intuitive impulses get in the way. Sometimes the latter are useful, sometimes they invite disaster. Modern economic thinking is shaping the view that decisions are often the result of the interaction between fast intuitive thinking and the attempt (often unsuccessful) to behave in a rational way. While neoclassic economics concentrated on rationalistic behavior, other branches as, e.g., the literature on learning in games (following Kandori et al., 1993; Young, 1993) focused on the study of behavioral rules of thumb. More recently, dual-process models from psychology (Epstein, 1994; Sloman, 1996; Strack and Deutsch, 2004; Evans, 2008; Alós-Ferrer and Strack, 2014) have received increasing attention in economics. These models postulate decision-process heterogeneity at the intra-individual level, that is, the interaction of more intuitive and more deliberative processes within a decision maker's mind.

Individual heterogeneity, however, remains an important topic. Across individuals, heterogeneity concerns whether each particular decision maker relies more or less on one or the other kind of process. To measure this dimension, a number of scales and questionnaires have been developed. Among them are the Rational-Experiential Inventory of Epstein et al. (1996), including its two subscales *Faith in Intuition* (FI) and *Need for Cognition* (NFC), and the three-item Cognitive Reflection Test (CRT) of Frederick (2005), recently expanded by Toplak et al. (2014) and Primi et al. (2015). A recent branch of the literature has investigated interindividual differences regarding faulty probability judgments (heuristics and biases) using these scales. Oechssler et al. (2009) and Hoppe and Kusterer (2011) find that higher test scores in the CRT are correlated with lower incidences of certain biases, e.g., the conjunction fallacy (Tversky and Kahneman, 1983). As argued by Toplak et al. (2011), low CRT scores might indicate a tendency to act on impulse and give an intuitive response. Alós-Ferrer and Hügelschäfer (2012, 2016) showed that higher scores in Faith in Intuition are associated with higher error rates aligned with certain heuristics, e.g., based on representativeness or reinforcement, but found no systematic relation between the CRT and FI.

This work continues the exploration of individual differences in faulty probability judgments and extends previous works by considering process data. The dual-process literature naturally relies on process data for the analysis of multi-process decisions, an approach which allows inferences which would be impossible with choice data only. The simplest kind of process data arises from response times. However, the heuristics-and-biases literature typically relies on decisions made on the basis of verbal descriptions, that is, on relatively complex, non-repeatable questions related to a more or less artificial situation (as for instance, the LINDA problem from Tversky and Kahneman, 1983). The use of response times in such a setting faces two main difficulties.

The first difficulty is that within-subject comparisons for a single question are not possible. However, precisely those are the standard for response-times studies. In many behavioral studies, decisions are made in paradigms which allow for repetition, sometimes even for a large number of trials for each individual participant. In these cases, one can compare the response times of different responses for the same individual, which allows predictions linked to the very nature of processes. For instance, if (in an extreme case) it is assumed that a certain response overwhelmingly follows from a certain intuitive process, while another response overwhelmingly follows from a more deliberative one, one would predict the first response to be on average faster, simply because intuitive processes are faster. In a typical description-based decision, however, a paragraph-long decision situation is presented, the participant makes a decision, and moves on to a different question. Hence there is a unique observation per participant, which is either correct or not. It is not possible to test hypotheses on the relative speed of different responses, because such comparisons would be confounded with personal characteristics. For instance, if a process-based model predicted errors to be faster than correct responses in a given situation, and even if this prediction were correct, one might obtain the opposite result if participants giving correct responses had higher cognitive abilities, and the latter were associated with faster response times for the given situation.

For instance, Achtziger and Alós-Ferrer (2014) study a paradigm where a reinforcement-based heuristic can conflict or be aligned with more rational decision making (optimization based on Bayesian updating of beliefs). The main predictions of the study (following the Dual-Process Diffusion Model, Alós-Ferrer, 2016) concern the relative speed of errors and correct responses *for a given individual*, i.e., a within-participant comparison. Those are testable because the paradigm allows for repetition, with 60 different decisions per participant, and hence one typically has multiple errors and multiple correct responses for a participant. In a paradigm with one decision per participant (say, measuring the CRT), errors and correct decisions can simply not be compared within participants.

The second difficulty is that, when measuring biases in probability judgments through standard decisions from description, response times are relatively long. In contrast, the dual-process literature focuses on rather short response times (a few seconds at most). Long response times (say, around half a minute) will always include some deliberation, and hence any response-time differences accruing from intrinsic differences among the decision processes involved are likely to be washed away (see, e.g., Myrseth and Wollbrant, 2016). However, this does not mean that long response times are useless. It is a well-established fact that decisions where the decision maker faces stronger tradeoffs, or is “closer to indifference,” are harder and result in longer response times (Dashiell, 1937; Mosteller and Nogee, 1951). This fact can be extended to longer response times, capturing the intuition that if one alternative is clearly preferred, a fast decision ensues, but if two alternatives are similarly desirable, an inner struggle results in a slower decision. Following this logic, longer response times should be considered evidence of longer deliberation due to opposed tendencies.

In view of these difficulties, our study focused on within-subject comparisons across different questions. To this purpose, we created a number of alternative versions of well-established questions. The logic is as follows. Many of the questions used to study biases in probability judgment pit the correct response against an intuitive alternative favored by a heuristic. For instance, in the LINDA question, an incorrect response is intuitively attractive because it is stereotype-consistent. The same is true for the items in the CRT, where an intuitive response conflicts with the correct one. To examine process data associated with the conflict, we created non-conflict versions of those questions. Depending on the content of the question, however, one ends with qualitatively different non-conflict items. In some cases it is possible to turn around the question in such a way that the intuitive process will remain active and favor the correct response. We refer to the resulting items as *alignment questions*, because both processes remain active but are aligned in terms of prescribed choices. In other cases, however, it is not possible to force the intuitive process to favor the correct response. The conflict can still be removed by shutting down the intuitive process (removing the cue on which it acts), creating a *neutral* version of the original question. In one extreme case, however, this manipulation was not possible, but it was still possible to create a non-conflict version where the heuristic points to the correct answer, but where the exact process (type of computation) underlying the deliberative process in the conflict version does not apply. The resulting altered item is called a *heuristic question*.

To the best of our knowledge, there is no systematic study on response times for this type of questions. Hence, the analysis in this article is novel but exploratory. We collected choice data in a laboratory environment where participants answered a series of standard questions regarding probability judgments, the original CRT of Frederick (2005), and additional items from the extended CRT of Toplak et al. (2014). Crucially, we measured response times for those decisions. Additionally, we included a number of questionnaires measuring personality differences, including the short version of the Rational-Experiential Inventory of Epstein et al. (1996) (comprising FI and NFC) and the Big Five (McCrae and Costa, 1985).

The paper proceeds as follows. Section 2 details the experimental design and describes the sample, the methods, and the natural hypotheses regarding response times. Section 3 presents some preliminary, descriptive results of correlational nature. Section 4 presents results for the (extended) CRT questions, including evidence on response times. Section 5 presents the results for behavioral biases, including the relation to the CRT and evidence on response times. Section 6 concludes.

## 2. Methods

### 2.1. Experimental design

We investigated decision processes by measuring both choices and response times for a series of incentivized context-embedded scenarios (“decisions from description”). We focused on two types of problems. First, we employed items from the Cognitive Reflection Test introduced by Frederick (2005) and further extended by Toplak et al. (2014). Second, we used a sample of questions tackling typical decision biases in the domain of belief updating and probabilistic judgment, capturing the conjunction fallacy (Tversky and Kahneman, 1983), base-rate neglect (Kahneman and Tversky, 1972; Fiedler, 2000; Erev et al., 2008), and the ratio bias (Kirkpatrick and Epstein, 1992; Denes-Raj and Epstein, 1994).

For both types of problems, questions are assumed to create a situation of conflict between an “intuitive” answer favored by a certain heuristic process and the (normatively) correct response. We complemented each question with a non-conflict version, hence creating several pairs of items. We developed three categories of non-conflict versions. For some of the questions, we created alignment versions where the intuitive answer and the normatively correct answer coincide. For others we created neutral versions where the heuristic does not apply, so that there is no intuitive first answer. Further, for one of the CRT questions we created a heuristic version where the heuristic points to the correct answer, but where the computation process leading to the correct answer in the conflict version does not apply.

Presenting two versions of the same question within one experiment might potentially direct the participants' attention to the deceitful property of these questions. To reduce this problem while keeping the rationale of the questions intact, the surface similarity between two paired items was reduced by using different contextual and numerical contents (see, e.g., De Neys et al., 2013). For the comparison of response times to be meaningful, we matched the length of the items for each pair (all items were translated to German as we relied on a sample of German-speaking participants). That is, we adapted the wording of the questions to guarantee that the number of sentences was always the same for each pair. Further, the number of words, characters, and syllables of the German translations did not differ by more than 10% across the questions of a given pair. To this aim, in some cases we made slight cosmetic changes to the wording of the questions taken from the literature.

Overall, our sample of questions comprised the following items: Two pairs from the classic CRT (Frederick, 2005), plus the third original CRT item (without a matched non-conflict version) to be able to compute a CRT score for each participant; two pairs from the extended CRT by Toplak et al. (2014) (for other questions it was not possible to create non-conflict versions); a quartet referring to the conjunction fallacy; three pairs referring to base-rate neglect; and one pair referring to the ratio bias.

In addition, we investigated several individual correlates of the reliance on intuitive vs. deliberative decision making: Faith in Intuition and Need for Cognition (Epstein et al., 1996), Actively Open-Minded Thinking (Baron, 1993) (respectively referred to as FI, NFC, and AOT hereafter), and the Big Five personality scales (McCrae and Costa, 1985). We also controlled for numerical literacy (Lipkus et al., 2001), gender, and individual swiftness.

### 2.2. Participants

Participants were recruited using ORSEE (Greiner, 2004), a standard online recruitment system for economic experiments which allows for random recruitment from a predefined subject pool. Participants were native German-speaking students from the University of Cologne (Germany), excluding students majoring in psychology or economics. We only considered native speakers due to our focus on response times, since those are critically related to participants' language skills for the text-based problems we used. In addition, our recruiting rules excluded participants who had previously participated in any experiment employing the CRT. A total of 158 participants (101 female; age range 18−44, mean 23.44) participated in exchange for performance-based payment plus a show-up fee of 4 Euros. Three further participants had to be excluded from data analysis because they did not comply with the instructions.

### 2.3. Procedure

The experiment was conducted at the Cologne Laboratory for Economic Research (CLER) using z-Tree (Fischbacher, 2007). Experimental procedures were in accordance with the ethical standards laid down in the 1964 Declaration of Helsinki and its later amendments, and also standard practices in experimental economics (e.g., no-deception rule). In agreement with the ethics and safety guidelines at the CLER, participants were all pre-registered in the laboratory through ORSEE and had given written informed consent regarding the laboratory's guidelines (no further informed consent is necessary for particular experiments). Potential participants were informed of their right to abstain from participation in the study or to withdraw consent to participate at any time without reprisal.

In a first phase, participants were asked 21 incentivized questions. Specifically, at the end of the experiment they received 0.50 Euro cent for each correct answer. These questions comprised the (extended) CRT (9 items), the conjunction fallacy (4 items), base-rate neglect (6 items), and the ratio bias (2 items). All but two of the CRT items had to be answered in open format. That is, participants were required to type their numerical response into a blank box. The remaining two CRT items and all other questions were multiple-choice items with two or more possible answers each.

To control for possible order effects, participants were randomly assigned to four different counterbalance conditions (pseudo-randomized question order).^1^ For each pair, half of the participants worked on the conflict version before the non-conflict version, whereas the other half started with the non-conflict version. In addition, for each participant, half of the item pairs were first shown in the conflict version and later in the non-conflict version, and vice versa for the other half of pairs. Further, the two items of each pair were separated by at least three other items.

In a second phase, participants worked on the 11 items of the numeracy scale (Lipkus et al., 2001). They were informed that the computer would randomly draw one of the 11 items at the end of the experiment, and that they would receive 0.50 Euro cent if their answer to the selected item was correct.

In a third phase, which was not incentivized, participants completed the self-report questionnaires. Those included FI and NFC (measured by means of the 10-item Rational-Experiential Inventory; Epstein et al., 1996), the Big Five Inventory-SOEP (15-item version; Gerlitz and Schupp, 2005), and AOT (7-item version by Haran et al., 2013). Participants rated questionnaire items by placing marks on continuous left-right scales ranging from 0 (“completely false”) to 10 (“completely true”). An exception was AOT, which was rated on a 7-point scale for each item. Since participants might have been exposed to the CRT items in their daily life (e.g., through the press or the internet), we also asked them to indicate whether they had previously seen each of the classic CRT items. Finally, the questionnaire comprised socio-demographic questions (gender, age, and native language).

No time limit was imposed; participants were free to use as much time as needed for the incentivized questions and the questionnaires. As a proxy for swiftness (see Cappelen et al., 2015), we measured the time it took participants to read the brief introductory instructions for phases one and two, and the time it took them to answer the questions about age, gender, and native language in phase three. The sum of these two measures (reading time and demographic answer time in seconds) was used to create an (inverse) index of swiftness.

Payment was computed at the end of the experiment. A session lasted about 50 min and average earnings were 12.24 Euros (*SD* = 1.28).

### 2.4. Basic hypotheses for response times

Our basic hypotheses concern the comparison of response times for paired conflict and non-conflict questions. Following a dual-process logic (e.g., the Dual-Process Diffusion Model of Alós-Ferrer, 2016), the response time for a question where there is a conflict between an intuitive and a deliberative process can be decomposed in two parts. First, the time needed for conflict detection and resolution. Second, the actual process time, that is, the time needed by the process which actually generates the response to do so. Let *D*_*C*_ be the expected time necessary for conflict detection and resolution in the presence of an actual decision conflict. Further, let *T*_*H*_ be the expected response time of the intuitive (heuristic) process, and let *T*_*U*_ > *T*_*H*_ be the expected response time of the deliberative (utilitarian) process (please note that, to simplify notation, all quantities are expected times).

Actual response time will be the sum of conflict detection and resolution time and process time. However, depending on conflict resolution, the process actually delivering the response might be either the intuitive or the deliberative one. Since we only observe one decision for a given participant, the expected response time is hence *D*_*C*_ + *T*_*H*_ or *D*_*C*_ + *T*_*U*_, depending on which process is selected. The problem, of course, is that the actually selected process is unobservable. If a large enough set of answers for a fixed question was observed, the total expected response time would be

$$\begin{array}{l} D_{C}+\Delta T_{H}+\left(1-\Delta \right)T_{U} \end{array}$$

where Δ is the probability that the intuitive process is the one actually delivering the response.

These considerations are useful to derive experimental hypotheses for the comparison of response times across questions. Consider an alignment question where the conflict has been removed because both processes prescribe the same answer. Two effects can be expected. First, the conflict detection and resolution time *D*_*C*_ will be reduced, since there is no actual conflict. Second, there will be an increase in the probability Δ that the faster, intuitive process is used, since there is no need to inhibit it (or, in other words, we have more observations of the type *D*_*C*_ + *T*_*H*_ than of the type *D*_*C*_ + *T*_*U*_). Both effects point in the same direction and deliver the following experimental hypothesis.

**H1**. Response times for alignment questions are shorter than response times for the analogous conflict questions.

Consider now a neutral question, where the intuitive process has been shut down by removing the cue on which it acts. The conflict detection and resolution time *D*_*C*_ will also be reduced in this case (absence of conflict). However, the probability that the intuitive process is actually used becomes Δ = 0. Hence response times will be shorter with respect to conflict detection but all decisions will arise from the slower, deliberative process. Evidence from neuroscience points out that conflict detection and resolution occurs extremely early in decision making (see, e.g., Achtziger et al., 2014) and hence should have a moderate effect in response times of large magnitude. In Achtziger and Alós-Ferrer (2014), decisions where a reinforcement heuristic had been shut down were observed to be significantly slower (and error rates significantly lower) than decisions where the heuristic was active. On the basis of this evidence, we formulate the following hypothesis.

**H2**. Response times for neutral questions are longer than response times for the analogous conflict questions.

However, alternative hypotheses might also be reasonable. Following the interpretation of long response times as evidence for deliberative struggle, one could speculate that the presence of conflict in decisions as the ones considered here has an effect beyond conflict detection and resolution. However, at this point there is no empirical basis for a comparison of the magnitude of this effect and the slowing-down of decisions in neutral questions due to the shutdown of the intuitive process.

In one case, the non-conflict question involves the intuitive process becoming prescriptively correct while the original deliberative process is shut down (heuristic question). In this case, again *D*_*C*_ should be reduced, and either the likelihood of the intuitive process being selected should become Δ = 1, or the deliberative process should be replaced with another, simpler and presumably faster one. In both cases, we would expect to observe faster decisions.

**H3**. Response times for heuristic questions are shorter than response times for the analogous conflict questions.

## 3. Descriptive results

### 3.1. Summary statistics and gender effects

Table 1 displays summary statistics for the main dependent variables and reports the presence or absence of gender differences (via Wilcoxon Rank-Sum tests on the whole sample). On average, participants correctly answered two out of the three classic CRT items by Frederick (2005), and one out of the two extended CRT items by Toplak et al. (2014). In line with previous studies (Frederick, 2005; Oechssler et al., 2009; Brañas-Garza et al., 2012; Alós-Ferrer and Hügelschäfer, 2016; Cueva et al., 2016), males had significantly higher scores in the classic three-item CRT; there was no difference concerning CRT2. The results regarding pre-experimental knowledge of the classic CRT imply that the test is becoming common knowledge in the student population: 13.92% of participants reported knowing one question, 26.58% two questions, and 36.08% all three. Participants with more previous knowledge of the items obtain significantly higher classic-CRT scores (Spearman's correlation, ρ = 0.307, *p* < 0.0001).

**Table 1:** **Summary statistics**.

**Variable**	**Mean**	**Min**.	**Max**.	**Mean (females)**	**Mean (males)**	**WRT for gender effects**
**NR. OF CORRECT ANSWERS**						
Total	16.475	10	22	16.149	17.053	*z* = −2.35, *p* = 0.019^**^
	(2.568)			(2.475)	(2.649)	
Classic CRT	2.025	0	3	1.851	2.333	*z* = −2.88, *p* = 0.004^***^
	(1.009)			(1.043)	(0.873)	
CRT2	1.38	0	2	1.366	1.404	*z* = −0.08, *p* = 0.936
	(0.683)			(0.717)	(0.623)	
Conjunction fallacy	3.076	1	4	3.05	3.123	*z* = −0.63, *p* = 0.529
	(0.878)			(0.865)	(0.908)	
Base-rate neglect	3.816	1	6	3.772	3.895	*z* = −0.83, *p* = 0.409
	(0.943)			(0.937)	(0.958)	
Ratio bias	1.892	1	2	1.871	1.93	*z* = −1.14, *p* = 0.256
	(0.311)			(0.337)	(0.258)	
Numeracy	9.652	5	11	9.386	10.122	*z* = −3.38, *p* = 0.001^***^
	(1.391)			(1.150)	(1.449)	
**PERSONALITY TRAITS**						
Faith in intuition	6.354	1.94	10	6.313	6.427	*z* = −0.44, *p* = 0.660
	(1.568)			(1.596)	(1.528)	
Need for cognition	6.096	2.08	9.84	5.905	6.435	*z* = −2.22, *p* = 0.027^**^
	(1.372)			(1.462)	(1.131)	
Openness to experience	5.764	1.333	9.467	5.808	5.685	*z* = 0.45, *p* = 0.651
	(1.861)			(1.849)	(1.895)	
Conscientiousness	6.391	0.333	10	6.359	6.449	*z* = −0.13, *p* = 0.901
	(1.889)			(1.857)	(1.96)	
Extraversion	6.289	0.433	10	6.549	5.829	*z* = 2.00, *p* = 0.046^**^
	(2.298)			(2.258)	(2.318)	
Agreeableness	6.850	2.1	10	6.774	6.985	*z* = −0.68, *p* = 0.500
	(1.729)			(1.718)	(1.755)	
Neuroticism	5.547	0	10	5.942	4.847	*z* = 2.87, *p* = 0.004^***^
	(2.251)			(2.024)	(2.473)	
Actively open-minded thinking	4.927	2.142	6	4.915	4.950	*z* = −1.25, *p* = 0.211
	(4.783)			(4.318)	(5.55)	
Known CRT items	1.753	0	3	1.653	1.93	*z* = −1.49, *p* = 0.137
	(1.177)			(1.178)	(1.163)	
Swiftness	94.546	36.941	169.449	95.178	93.426	*z* = 0.48, *p* = 0.631
	(29.205)			(28.815)	(30.110)	

Nr. of Correct Answers only refers to the conflict versions of the questions. The variable Classic CRT refers to the three original items of Frederick (2005). The variable CRT2 refers to the two extended-CRT conflict questions taken from Toplak et al. (2014). Numbers in parentheses are standard deviations.

Descriptive statistics for the numeracy scale (Lipkus et al., 2001) suggest that this measure is not particularly well-suited to capture interindividual differences. It exhibits a very low variance, with most of our participants answering either 10 or 11 out of 11 items correctly. Still, there is a significant gender difference, pointing to higher numeracy for males. Regarding personality traits, we find higher values of NFC for male compared to female participants, in line with previous research (Pacini and Epstein, 1999). Female participants have higher scores for Extraversion and Neuroticism, which is consistent with the literature (e.g., Feingold, 1994; Weisberg et al., 2011).

### 3.2. Personality measures

Table 2 displays Spearman rank correlations among personality traits. We include numerical literacy, but this measure shows no correlation with any of the personality traits. In contrast to theoretical assumptions of the Rational-Experiential Inventory (REI) (Epstein, 1994; Epstein et al., 1996), there is a weak positive correlation between FI and NFC in our sample (Spearman's correlation, ρ = 0.14, *p* = 0.079). Concerning the relation between the REI and the Big Five, we found that FI is positively associated with Openness to Experience, Conscientiousness, and Extraversion, while NFC is positively correlated with Openness to Experience and Conscientiousness, and negatively with Neuroticism. These results are perfectly consistent with the findings of Pacini and Epstein (1999). The significant positive correlations of AOT with NFC and Openness are in line with results by Haran et al. (2013).

**Table 2:** **Spearman correlations among personality traits (and numeracy)**.

	**1. Num**	**2. FI**	**3. NFC**	**4. Open**	**5. Consc**	**6. Extra**	**7. Agree**	**8. Neuro**
2. FI	0.067	–						
3. NFC	−0.107	0.140^*^	–					
4. Open	0.102	0.443^***^	0.190^**^	–				
5. Consc	0.047	0.163^**^	0.246^**^	0.034	–			
6. Extra	−0.038	0.261^***^	0.053	0.165^**^	0.154	–		
7. Agree	0.129	0.034	−0.030	0.130	0.090	0.043	–	
8. Neuro	−0.057	−0.072	−0.226^**^	−0.065	−0.065	−0.223^***^	0.033	–
9. AOT	0.049	0.062	0.266^***^	0.191^**^	0.074	−0.003	0.226^***^	−0.003

Num, Numeracy; FI, Faith in intuition; NFC, Need for cognition; Open, Openness to experience; Consc, Conscientiousness; Extra, Extraversion; Agree, Agreeableness; Neuro, Neuroticism; AOT, Actively open-minded thinking.

* p < 0.10,

** p < 0.05,

*** p < 0.01.

## 4. Extended CRT questions

For the analysis of response times, in a first step we removed outliers in order to exclude abnormal observations that might bias the results. To this end, we removed, for each item, response times that deviated more than two standard deviations from the respective mean of the whole sample of participants (see Miller, 1991, on this). This led to the exclusion of several very slow responses, but not of very fast ones. Further, we excluded response times of zero, which resulted from a few participants accidentally skipping a question by double-clicking. Hence, for every paired-observations test across the two questions in a pair, participants whose response times were outliers in either of the two questions are removed. In order to test our hypotheses on response times, we use non-parametric, two-tailed Wilcoxon Signed-Rank tests (for paired observations). To compare error rates across the two questions in a pair, we rely on McNemar's chi-squared test, which is based on the number of discordant pairs. For ease of presentation, instead of repeating the exclusion criteria for every single item pair, we report for each test the corresponding *N*, that is, the number of participants with valid response times in both of the two questions. The number of exclusions for each test is simply the difference between the reported number of observations and the total sample size of *N* = 158.

### 4.1. Question-level analysis

In the following subsections we present the CRT questions used in the present study, together with the corresponding analyses of error rates and response times of the matched pairs. For each pair we briefly outline the rationale behind the conflict version and the construction of the non-conflict version. Given the frame modification that some of the original CRT questions underwent to minimize recognizability, we report the text (English translation of the German items) also for those original CRT questions.

#### 4.1.1. The bat and the ball: conflict vs. heuristic

The first pair of questions presented corresponded to the famous “bat and the ball” problem (Frederick, 2005). A non-conflict version of this question has been previously studied by De Neys et al. (2013) and Johnson et al. (2016).

**(Q1C)** A postcard and a pen cost 110 cents in total. The postcard costs 100 cents more than the pen. How much does the pen cost? (In cents)
  [correct answer = 5]
**(Q1H)** A magazine and a banana together cost 290 cents. The magazine costs 200 cents. What is the price of the banana? (In cents)
  [correct answer = 90]

For the classic (Q1C) question, there is an intuitive but wrong answer (“10”). This presumably involves participants focusing on the numbers, quickly segmenting the 110 cents into 100 and 10 cents, thereby neglecting the “more than” statement. Question (Q1H) provides a control version of the problem, developed by De Neys et al. (2013). By eliminating the words “more than” from the question, it allows the intuitive segmentation mechanism to produce the correct answer. At the same time, however, the computation process that provides the correct solution in (Q1C) cannot be applied in this problem anymore. It becomes entirely inappropriate, since the solution is transparent. Hence this non-conflict version of the question (which, to the best of our knowledge, follows the obvious way to remove the conflict), neither generates process alignment nor shuts down the intuitive process. Rather, it corresponds to the *heuristic question* case we have described above.

**Figure 1:**
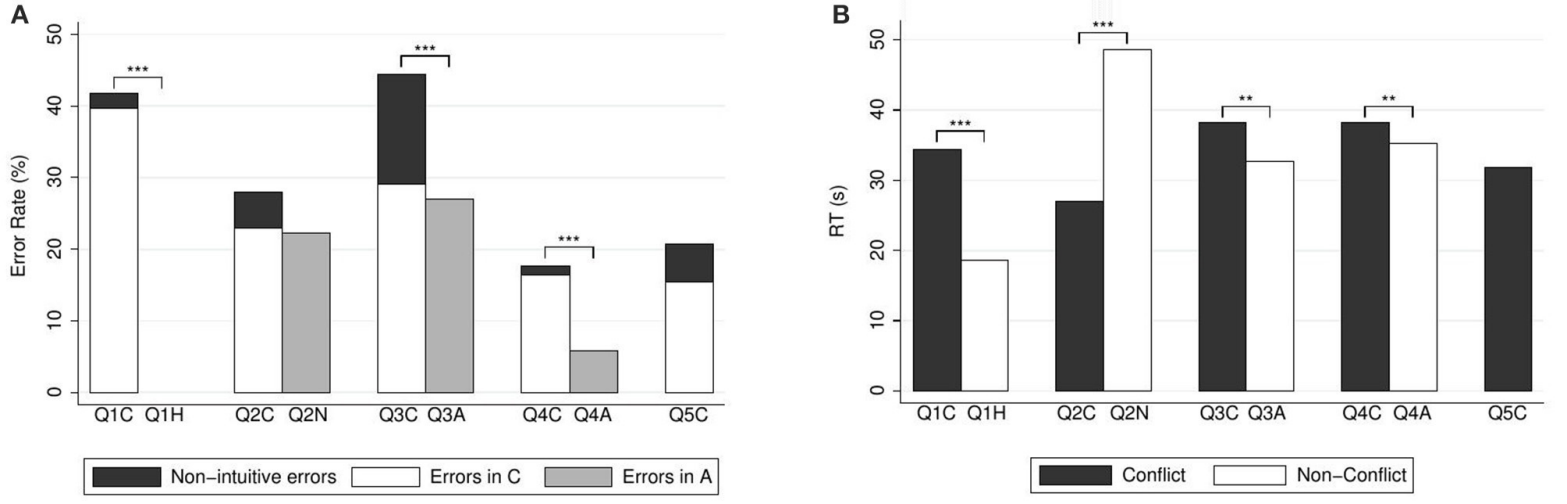
**(A)** Error rates (in %) and **(B)** mean response times (in seconds) for CRT questions. Reported significance refers to McNemar's tests for error rates and to Wilcoxon Signed-Rank tests for response times. ^**^p < 0.05, ^***^p < 0.01.

Figure 1 depicts the percentages of errors and correct responses (panel A) and the response times (panel B). Participants' answers to the conflict question were significantly slower than their answers to the heuristic question (median response time 29.14 s, mean 34.38 s, *SD* = 22.00 in case of conflict; median 17.29 s, mean 18.71 s, *SD* = 6.42 for the heuristic question; WSR test, *N* = 141, *z* = 7.55, *p* < 0.001). This is consistent with hypothesis H3.

There were significantly more errors in the conflict question than in the heuristic version. For (Q1C), there were 39.72% (56) heuristic errors, 2.13% (3) non-heuristic errors (responses other than five or ten), and 58.16% (82) correct answers. For (Q1H), all answers were correct. Unsurprisingly, the proportion of errors in the conflict question was significantly larger than in the heuristic question [McNemar's test, *N* = 141, $\chi_{\left(1\right)}^{2}=59.00$, *p* < 0.001].

#### 4.1.2. Making widgets: conflict vs. neutral

The second pair of questions again corresponds to one of the classic CRT items of Frederick (2005). The non-conflict version corresponds to our neutral category.

**(Q2C)** If it takes 5 machines 5 minutes to make 5 car tires, how long would it take 100 machines to make 100 car tires? (In minutes)
  [correct answer = 5]
**(Q2N)** If it takes 60 machines 100 minutes to make 60 bricks, how long would it take 100 machines to make 100 bricks? (In minutes)
  [correct answer = 100]

The number repetition in (Q2C) induces many participants to complete the pattern and give the intuitive but wrong answer “100.” (Q2N) provides a control version where the pattern is broken. By excluding the possibility of recognizing and reproducing a simple pattern, (Q2N) excludes the possibility of using a heuristic shortcut as in (Q2C). However, the same computation process that provides the correct solution in the conflict version can still be applied in this problem. Therefore, (Q2N) is a neutral counterpart of the conflict item (Q2C).

Figure 1 depicts the percentages of errors and correct responses (panel A) and the response times (panel B). Answers to the conflict question were significantly faster than the answers to the neutral question (median response time 21.61 s, mean 27.08 s, *SD* = 18.10 in case of conflict; median 37.16 s, mean 48.69 s, *SD* = 33.07 for the neutral question; WSR test, *N* = 139, *z* = −7.68, *p* < 0.001). This is in agreement with our Hypothesis H2.

Regarding choice data, for (Q2C) there were 23.02% (32) heuristic errors, 5.04% (7) non-heuristic errors, and 71.94% (100) correct answers. For (Q2N), there were 22.30% (31) errors, and 77.70% (108) correct answers. According to McNemar's test, the proportion of errors in the conflict question was not significantly different than in the neutral question [*N* = 139, $\chi_{\left(1\right)}^{2}=2.29$, *p* = 0.131]. Please note, however, that throughout the paper we rely on two-sided tests. If we used a one-sided test here (based on our directional prediction), the result would of course be (marginally) significant.

#### 4.1.3. Buying and selling: conflict vs. alignment

The third pair of questions we used was taken from the extended-CRT questions of Toplak et al. (2014), for which we developed an alignment version.

**(Q3C)** A man buys a pig for 60 Euros, sells it for 70 Euros, buys it back for 80 Euros, and finally sells it for 90 Euros. How much has he made? (In Euros)
  [correct answer = 20]
**(Q3A)** A man buys a cow for 60 Euros, sells it for 70 Euros, buys it back for 70 Euros, and finally sells it for 90 Euros. How much has he made? (In Euros)
  [correct answer = 30]

For the (Q3C) question, there is an intuitive but wrong answer (“10”). This is due to a miscalculation of the earnings where the difference between each two consecutive buying or selling actions is computed, instead of computing the profits or losses from every buy-and-sell operation. That is, participants compute (70−60)+(70−80)+(90−80) = 10 instead of (70−60)+(90−80) = 20. In (Q3A), by having equal numbers in the middle of the question, this heuristic but incorrect way of thinking provides the correct answer. Importantly, the computation process that provides the correct solution is the same in (Q3C) and (Q3A). Therefore (Q3A) is an alignment counterpart of the conflict item (Q3C).

Figure 1 depicts the percentages of errors and correct responses (panel A) and the response times (panel B). Answers to the conflict question were significantly slower than the answers to the alignment question (median response time 33.74 s, mean 38.23 s, *SD* = 18.40 in case of conflict; 28.92 s, mean 32.66 s, *SD* = 15.03 in case of alignment; WSR test, *N* = 144, *z* = 2.42, *p* = 0.015). This is in agreement with our Hypothesis H1.

Alignment of course produces a simpler question, since the intuitive process becomes a cognitive shortcut. It was hence expected that there would be less errors under alignment. For (Q3C), there were 29.17% (42) heuristic errors, 15.27% (22) non-heuristic errors, and 55.56% (80) correct answers. For (Q3A), there were 27.08% (39) errors, and 72.92% (105) correct answers. According to McNemar's test, the proportion of errors in the conflict question was significantly larger than in the alignment question [*N* = 144, $\chi_{\left(1\right)}^{2}=15.24$, *p* < 0.001].

#### 4.1.4. Up and down: conflict vs. alignment

The fourth pair of questions presented to participants is the seventh item in the list of extended CRT questions by Toplak et al. (2014), which follows a multiple-choice format. Our non-conflict version follows one developed by Bieleke and Gollwitzer for a different purpose (manuscript in preparation).

**(Q4C)** In January, Lucas invested 8000 Euros in the stock market. In July, the stocks he had purchased were down 50%. Fortunately, from July to October, they went up 75%. In October, Lucas
  ◦ has broken even in the stock market.
  ◦ is ahead of where he began.
  ◦ has lost money.
  [correct answer = has lost money]
**(Q4A)** On Monday, the temperature was 22°C in the shade. Two days later, the temperature was down by 50%. Fortunately, the temperature went up 125% again by Saturday. Compared to Monday, the temperature on Saturday is
  ◦ warmer.
  ◦ the same.
  ◦ colder.
  [correct answer = warmer]

In this problem, participants typically focus on the fact that the later percentage increase is larger than the earlier percentage decrease, neglecting that the amount to which the increase is applied is not the starting amount. Hence, many participants erroneously select the second option in (Q4C). In (Q4A), by making the percentage increase larger, the heuristic shortcut provides the correct answer even if the way of thinking is erroneous. Still, the correct answer can also be reached by means of the same computation mechanism that is required to correctly answer (Q4C). Therefore, (Q4A) is an alignment counterpart of the conflict item (Q4C).

Figure 1 depicts the percentages of errors and correct responses (panel A) and the response times (panel B). Answers to the conflict question were significantly slower than the answers to the alignment question (median response time 35.95 s, mean 38.32 s, *SD* = 15.06 in case of conflict; median 33.24 s, mean 35.25 s, *SD* = 13.50 in case of alignment; WSR test, *N* = 152, *z* = 2.25, *p* = 0.024). Again, this is in agreement with our Hypothesis H1.

As in the previous pair, there were significantly less errors under alignment. For (Q4C), there were 16.45% (25) heuristic errors, 1.32% (2) non-heuristic errors, and 82.24% (125) correct answers. For (Q4A), there were 5.92% (9) errors, and 94.08% (143) correct answers. According to McNemar's test, the proportion of errors in the conflict question was significantly larger than in the alignment question [*N* = 152, $\chi_{\left(1\right)}^{2}=9.00$, *p* = 0.003].

#### 4.1.5. Growing in the lake

In order to be able to compute the standard CRT score, we also included the last of the classic three CRT items of Frederick (2005).

**(Q5C)** In a lake, there is a patch of lily pads. Every day, the patch doubles in size. If it takes 48 days for the patch to cover the entire lake, how long would it take for the patch to cover half of the lake? (In days)
  [correct answer = 47]

For this question, there is an intuitive but wrong answer (“24”), produced by halving the number of days, ignoring the exponential growth of the lily pads. The structure of the question makes it impossible to create a non-conflict version without making it exceedingly trivial. Hence, this item was not paired with a non-conflict version.

After removing response-time outliers, our sample for the (Q5C) question contains *N* = 149 observations. The median response time was 27.33 s, mean 31.85 s, *SD* = 16.79 (Figure 1B). There were 15.44% (23) heuristic errors, 5.37% (8) non-heuristic errors, and 79.19% (118) correct answers (Figure 1A).

### 4.2. Regression analysis for extended CRT questions

Our data forms a perfectly balanced panel with 9 decisions per participant. Hence we rely on random-effects panel regressions. This allows us to control for a variety of variables that might affect choices or response times, such as the number of words and letters and participants' swiftness.

Table 3 displays the results of panel regressions for response times. Contrary to the individual tests, we did not drop participants with outlier response times. Dropping those participants would have greatly reduced the sample since the regression covers all questions simultaneously. Instead, we relied on random effects and a log-transformation of response times (and controlling for swiftness). We only had to drop one of the participants from the whole sample because he left one of the answers blank. Model 1 contains dummies for the different versions of questions (heuristic, neutral, and alignment; conflict is the reference category). All dummies are significant, implying longer response times for neutral questions and shorter response times for heuristic and alignment questions, in agreement with Hypotheses H1, H2, and H3. Further, they remain significant when controlling for interindividual heterogeneity (Model 2). Not surprisingly, previous knowledge of the classic CRT items reduces response times.

**Table 3:** **Random-effects regressions on log response times of CRT questions**.

**Log(response time)**	**Model 1**	**Model 2**	**Model 3**
Heuristic	−0.438^***^	−0.438^***^	−0.356^***^
	(0.075)	(0.075)	(0.073)
Neutral	0.443^***^	0.443^***^	0.482^***^
	(0.158)	(0.159)	(0.157)
Alignment	−0.063^*^	−0.063^*^	−0.002
	(0.037)	(0.038)	(0.035)
Number of words	0.006	0.006	0.006
	(0.011)	(0.011)	(0.010)
Number of letters	0.001	0.001	0.001
	(0.001)	(0.001)	(0.001)
Log(swiftness)		0.101^*^	0.103^*^
		(0.053)	(0.061)
Numeracy		−0.040	−0.026
		(0.033)	(0.030)
Male		0.057	0.053
		(0.058)	(0.053)
Known CRT items		−0.114^***^	−0.109^***^
		(0.036)	(0.037)
Faith in intuition		−0.006	−0.007
		(0.016)	(0.016)
Need for cognition		−0.020	−0.018
		(0.018)	(0.020)
Openness to experience		−0.005	−0.006
		(0.016)	(0.016)
Conscientiousness		−0.022^***^	−0.021^***^
		(0.006)	(0.007)
Extraversion		0.022^***^	0.020^***^
		(0.003)	(0.003)
Agreeableness		0.014	0.017
		(0.010)	(0.012)
Neuroticism		0.009	0.006
		(0.010)	(0.010)
Actively open-minded thinking		0.022	0.020
		(0.028)	(0.033)
Non-intuitive error			0.458^***^
			(0.027)
Intuitive error			0.167^***^
			(0.059)
Intuitive error × alignment			−0.163^**^
			(0.073)
Observations	1413	1413	1413

The dummies Heuristic, Neutral, and Alignment take the value 1 for the respective versions of the questions; Conflict is the reference category. Standard errors (in parentheses) are (conservatively) clustered at the level of counterbalance condition (question order).

* p < 0.1,

** p < 0.05,

*** p < 0.01.

In Model 3, we introduce dummies for intuitive and non-intuitive errors. The results imply that participants making non-intuitive errors are slower. A *post-hoc* test further shows that participants making an intuitive error under conflict are significantly slower than those giving the correct answer under conflict (coefficient 0.167, *SD* = 0.060, *z* = 2.84, *p* = 0.004). However, there was no difference between those committing an error and those giving a correct answer under alignment (*post-hoc* test, coefficient 0.004, *SD* = 0.044, *z* = 0.10, *p* = 0.923). In any case, this should not be confused with a statement on the relative speed of errors, which would be a within-subject comparison. Since this is a comparison across subjects, it merely points out that participants giving incorrect answers might be cognitively slower than participants giving correct answers.

We now turn to random-effects probit panel regressions on correct answers to the CRT questions (Table 4). As expected, the likelihood of a correct answer is higher in the absence of conflict, as reflected by a dummy pooling alignment, neutral, and heuristic questions. Participants scoring high in numeracy are more likely to answer the CRT questions correctly, in spite of the low variance in this scale. There is also a gender effect, with males providing correct responses more often. However, once FI, NFC, and numerical literacy are included (Model 2), the gender difference disappears. Effects remain significant when controlling for further heterogeneity, including the Big Five and AOT (Model 4).

**Table 4:** **Random-effects probit regressions on correct answers to CRT questions**.

**Correct answer**	**Model 1**	**Model 2**	**Model 3**	**Model 4**
Male	0.259^**^	0.119	0.118	0.026
	(0.123)	(0.144)	(0.144)	(0.130)
Non-conflict	0.632^***^	0.629^***^	0.931^**^	0.923^**^
	(0.027)	(0.027)	(0.373)	(0.377)
Faith in intuition (FI)		−0.007	−0.018	0.006
		(0.016)	(0.017)	(0.019)
Need for cognition (NFC)		0.010	0.039	0.026
		(0.033)	(0.041)	(0.036)
Numeracy		0.186^***^	0.185^***^	0.187^***^
		(0.034)	(0.033)	(0.033)
Non-conflict × FI			0.029	0.031
			(0.026)	(0.027)
Non-conflict × NFC			−0.080	−0.081
			(0.063)	(0.063)
Openness to experience				−0.051^*^
				(0.030)
Conscientiousness				0.030
				(0.035)
Extraversion				−0.022
				(0.023)
Agreeableness				0.071^***^
				(0.022)
Neuroticism				−0.053^***^
				(0.012)
Actively open-minded thinking				−0.009
				(0.048)
Observations	1413	1413	1413	1413

The dummy Non-Conflict subsumes alignment, neutral, and heuristic problems; Conflict is the reference category. Standard errors (in parentheses) are (conservatively) clustered at the level of counterbalance condition (question order).

* p < 0.1,

** p < 0.05,

*** p < 0.01.

### 4.3. Discussion: extended CRT questions

Response times are typically in the 15−35 s range, which is considerably longer than response times studied in the dual-process literature. Hence, it is clear that practically all decisions involve deliberation and no relevant part of the observations can be viewed exclusively as the result of a fast, automatic (intuitive or heuristic) process in the sense of the dual-process literature (Epstein, 1994; Strack and Deutsch, 2004). However, a mechanistic interpretation of process conflict and alignment, as given in Section 2.4, might still help organize and understand the data.

The predictions derived from this interpretation (Section 2.4) were clearly supported by the data. Overall, the study of response times related to CRT questions suggests that even at this long time scale, this kind of questions fall well within the domain of dual-process theories. It is conceptually useful to identify behavioral tendencies with decision processes and consider intuitive ones as more automatic (hence faster) processes.

When analyzing response times, a large individual heterogeneity has to be expected, and differences will become more important at longer time scales. The regression analysis confirmed our basic findings while controlling for individual differences, including a number of personality factors, and an individual measure of swiftness (which was, as expected, significant). To study actual choices, we also conducted probit regressions on individual answers, which showed the expected effects, e.g., non-conflict question versions were easier.

The regressions also allowed us to examine the influence of personality factors on behavior. Interestingly, scales as FI, NFC, and AOT had no impact on CRT questions (neither on answers nor on response times), in agreement with previous evidence that these measures appear to diverge greatly at the individual level (Alós-Ferrer and Hügelschäfer, 2016). However, the inclusion of these measures eliminates apparent gender effects, pointing out that gender differences in performance in CRT-style questions might be explained by personality differences correlated with gender. Regarding the Big-Five Inventory, Conscientiousness led to faster responses and Extraversion to slower ones, but neither had a significant effect on responses. Agreeableness led to significantly more correct answers and Neuroticism and Openness to Experience to more errors, but none of them had a significant effect on response times.

Finally, it is important to note that the items we have considered (as the ones related to decision biases analyzed in the next section) belong to the category of *decisions from inference*, in the sense that there is an objectively correct answer which needs to be identified. This is in contrast to *preferential choice*, where by definition there is no objectively correct response (for example, consider lottery-choice questions). For decisions from inference, it is in principle possible to derive natural hypotheses on the nature of the involved processes in advance, as our discussions above illustrate. For preferential choice, the picture is less clear, because the very nature of the involved processes is part of the research question. We will return to this point in the discussion.

## 5. Decision biases

In the following subsections we present the questions capturing the decision biases investigated in the present study, together with the corresponding analyses of error rates and response times of the matched items. For each question we report the English translation of the German items we used, and briefly outline the rationale behind the conflict version and the construction of the non-conflict version. The same criteria for outliers and tests were used as in Section 4.

To explore the influence of personality factors, we also test whether participants' proneness to decision biases is related to their CRT, FI, and NFC scores, following Alós-Ferrer and Hügelschäfer (2012, 2016). In particular, Alós-Ferrer and Hügelschäfer (2016) observed that higher CRT scores were linked to a lower likelihood of committing the conjunction fallacy and base-rate neglect, in line with previous research (Oechssler et al., 2009; Hoppe and Kusterer, 2011). Similarly, lower FI scores were associated with a lower likelihood of these biases, albeit not as consistently as CRT scores. Whereas those results were based on median splits, we ran random-effects probit regressions in order to take advantage of the full range of scores, regressing correct answers to the conflict versions of the bias questions on participants' CRT, FI, and NFC score. We defined the additional variable CRT2 as the score in the two additional conflict items taken from Toplak et al. (2014) (hence, CRT2 can take the values 0, 1, or 2). Results are shown in Table 5 and are discussed in the respective subsections below.

**Table 5:** **Random-effects probit regressions on correct answers to decision-bias questions**.

**Correct answer**	**BRN1**	**BRN2**	**BRN3**	**Conj. fall**.	**Ratio bias**
Classic CRT	0.535	−0.025	0.320^**^	0.379^***^	0.513^***^
	(0.764)	(0.109)	(0.144)	(0.113)	(0.161)
CRT2	0.708	0.211	0.401^*^	−0.113	−0.138
	(1.112)	(0.165)	(0.216)	(0.169)	(0.237)
Faith in intuition	0.198	0.133^*^	0.036	0.070	0.088
	(0.508)	(0.069)	(0.085)	(0.070)	(0.102)
Need for cognition	0.246	−0.035	0.102	−0.054	−0.144
	(0.543)	(0.083)	(0.102)	(0.081)	(0.117)
Observations	158	158	158	158	158

Correct Answer refers to the conflict version of the respective questions. BRN1-3 indicate the three base-rate-neglect problems. Standard errors (in parentheses) are (conservatively) clustered at the level of counterbalance condition (question order).

* p < 0.1,

** p < 0.05,

*** p < 0.01.

### 5.1. Base-rate neglect

The first group of questions on decision biases refers to base-rate neglect. This phenomenon occurs when decision makers overweight sample information at the expense of the base rate. To examine this bias, we used three pairs of questions.

#### 5.1.1. Taxicabs and base-rate neglect: conflict vs. alignment

The first question is the celebrated “Taxicab question” from Kahneman and Tversky (1972), studied by Tversky and Kahneman (1980) and Bar-Hillel (1980), which we implemented as a multiple-choice problem.

**(BR1C)** In a city there are two cab companies, the Green and the Blue. 85% of the cabs in the city are Green and 15% are Blue. A cab was involved in a hit-and-run accident last night. A witness identified the cab as a Blue cab. The court tested his ability to distinguish between Green and Blue cabs at night. The witness made correct identifications in 80% of the cases and erred in 20% of the cases. The probability that the cab involved in the accident was Blue rather than Green is
  ◦ larger than 50%.
  ◦ smaller than 50%.
**(BR1A)** In a city there are two limousine companies, the Yellow and the Pink. 60% of the limousines in the city are Yellow and 40% are Pink. A limousine was involved in a hit-and-run accident last night. A witness identified the limousine as a Yellow. The court tested his ability to distinguish between Yellow and Pink limousines at night. The witness made correct identifications in 70% of the cases and erred in 30% of the cases. The probability that the limousine involved in the accident was Yellow rather than Pink is
  ◦ larger than 50%.
  ◦ smaller than 50%.

Bayes' Rule yields a posterior probability of ~41% in the conflict version (BR1C), and a probability of ~78% in the alignment version (BR1A). However, in studies involving probability estimates, median answers in (BR1C) are typically around 80% (e.g., Bar-Hillel, 1980). This is because decision makers typically underweight the base rate, and their answers are dominated by the witness' credibility instead. Hence, for (BR1C), the intuitive but normatively wrong answer is to choose the first option (larger than 50%). In (BR1A), by increasing the base rate, the same heuristic that misled participants in (BR1C) now provides the correct answer. Therefore (BR1A) is an alignment counterpart of the conflict item (BR1C). We remark that Bar-Hillel (1980) developed a different and more extreme non-conflict version, but we developed our own because in that version, the statement of a witness is replaced by more specific information which actually dominates the base rate, so that neglecting the base rate is appropriate.

**Figure 2:**
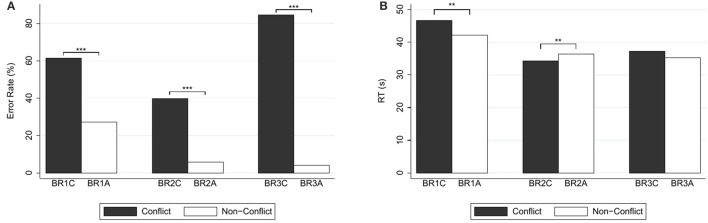
**(A)** Error rates (in %) and **(B)** mean response times (in seconds) for base-rate-neglect questions. Reported significance refers to McNemar's tests for error rates and to Wilcoxon Signed-Rank tests for response times. ^**^p < 0.05, ^***^p < 0.01.

Figure 2 depicts the percentages of errors and correct responses (panel A) and the response times (panel B). Answers to the conflict question were significantly slower than the answers to the alignment question (median response time 43.73 s, mean 46.78 s, *SD* = 19.13 in case of conflict; 38.70 s, mean 42.23 s, *SD* = 14.43 in case of alignment; WSR test, *N* = 146, *z* = 2.01, *p* = 0.044), in agreement with our basic Hypothesis H1.

Regarding error rates, for the conflict question (BR1C), there were 61.64% (90) errors, and 38.36% (56) correct answers. For (BR1A), there were 27.40% (40) errors, and 72.60% (106) correct answers. As is to be expected for a comparison between a conflict and an alignment version, the proportion of errors in the conflict question was significantly larger than in the alignment question [McNemar's test, *N* = 146, $\chi_{\left(1\right)}^{2}=27.17$, *p* < 0.001].

Last, we report on the relation to the CRT and the FI and NFC scales. Alós-Ferrer and Hügelschäfer (2016) found that the CRT had no informative value for the base-rate fallacy as captured by this particular question. We obtain the same null result. There were no effects of CRT score, FI or NFC on the likelihood of correctly answering the conflict version of this question (BR1C) (see Table 5).

#### 5.1.2. Detecting criminals and base-rate neglect: conflict vs. alignment

The next pair of questions used to measure base-rate neglect is analogous to a classic problem from Eddy (1982) and has been used by Hoppe and Kusterer (2011) and Alós-Ferrer and Hügelschäfer (2016).

**(BR2C)** In a city with 10 criminals and 10,000 innocent citizens there is a surveillance camera with an automatic face recognition software. If the camera sees a known criminal, it will trigger the alarm with 70% probability; if the camera sees an innocent citizen, it will trigger the alarm with a probability of 30%. The probability that indeed a criminal was filmed when the alarm is triggered is
  ◦ larger than 50%.
  ◦ smaller than 50%.
**(BR2A)** In a prison with 9000 criminals and 90 innocent people appeals to court take place regularly. When an appeal happens, it will sentence guilty a criminal with 75% probability; if the trial involves an innocent citizen, it will sentence him guilty with a probability of 25%. The probability that indeed a criminal was sentenced if the appeal judged him guilty is
  ◦ larger than 50%.
  ◦ smaller than 50%.

The posterior probability in the (BR2C) question is only ~23%, but due to base-rate neglect participants typically overweight the reliability of the test. Hence, the intuitive but incorrect response is to select the first option. In (BR2A), by increasing the base rate to put it in agreement with the diagnostic information, the same heuristic that misled participants in (BR2C) now provides the correct answer. Therefore (BR2A) is an alignment counterpart of the conflict item (BR2C).

Figure 2 depicts the percentages of errors and correct responses (panel A) and the response times (panel B). Answers to the conflict question were significantly *faster* than the answers to the alignment version (median response time 30.63 s, mean 34.38 s, *SD* = 15.95 in case of conflict; 34.80 s, mean 36.45 s, *SD* = 11.77 in case of alignment; WSR test, *N* = 138, *z* = 2.08, *p* = 0.037). This is inconsistent with the results for the previous question pair and with our Hypothesis H1.

Error rates, however, do not suggest a qualitative difference with the previous pair. For (BR2C) there were 39.86% (55) errors, and 60.14% (83) correct answers. For (BR2A) there were 5.80% (8) errors, and 94.20% (130) correct answers. According to McNemar's test, the proportion of errors in the conflict question was significantly larger than in the alignment question [*N* = 138, $\chi_{\left(1\right)}^{2}=37.44$, *p* < 0.001].

As shown in Table 5, participants' CRT score did not affect the likelihood of a correct answer to the (BRC2) item, in contradiction with the results by Hoppe and Kusterer (2011) and Alós-Ferrer and Hügelschäfer (2016). In the same way, NFC was not predictive for this item. FI level was predictive, but not in the expected direction (marginally significantly higher likelihood of answering correctly with higher FI level).

#### 5.1.3. Genetic disorders and base-rate neglect: conflict vs. alignment

The third pair of items is based on the original question of Eddy (1982).

**(BR3C)** Jonathan has been tested for a rare genetic disorder at his doctor. Only one in 10,000 people have this disorder. The test has very high detection rate: 99%. That means if Jonathan has the disorder, there is a 99% chance that the test is positive. The test also has a very low false-positive rate: 1%. That means that if Jonathan does not have the disorder, there is only a 1% chance that the test is positive. Unfortunately, Jonathan has tested positive for this disorder. The probability with which Jonathan has the genetic disorder is
  ◦ larger than 50%.
  ◦ smaller than 50%.
**(BR3A)** A doctor has tested a population of obese patients for high cholesterol in the blood. 8000 in 10,000 obese people have high cholesterol. The test has a very high detection rate: 95%. That means if a patient has high cholesterol, there is a 95% chance that the test is positive. The test also has a very low false-positive rate: 5%. That means that if a patient does not have high cholesterol, there is only a 5% chance that the test is positive. A patient has been tested positive for this condition. The probability with which the patient has high cholesterol is
  ◦ larger than 50%.
  ◦ smaller than 50%.

The logic here is the same as for (BR2C) and (BR2A). The posterior probability in the (BR3C) question is only around ~1%, but participants are tempted to select the first option. Due to the altered base rate, (BR3A) is an alignment counterpart of the conflict item (BR3C).

Figure 2 depicts the percentages of errors and correct responses (panel A) and the response times (panel B). Response times of answers to the conflict question were not significantly different from those to the alignment question (median response time 32.32 s, mean 37.22 s, *SD* = 18.47 in case of conflict; median 31.57 s, mean 35.38 s, *SD* = 14.77 in case of alignment; WSR test, *N* = 144, *z* = 0.62, *p* = 0.536). Hence, with respect to our Hypothesis H1, we cannot reject the null hypothesis of no differences in this case.

As in the case of the previous pair of questions, however, choice data reflect the normal difference between a conflict and an alignment version. For (BR3C), there were 84.97% (122) errors, and 15.28% (22) correct answers. For (BR3A), there were 4.17% (6) errors, and 95.83% (138) correct answers. According to McNemar's test, the proportion of errors in the conflict question was significantly larger than in the alignment question [*N* = 144, $\chi_{\left(1\right)}^{2}=114.03$, *p* < 0.001].

A higher CRT score significantly increased the likelihood of giving a correct answer to (BR3C) (see Table 5). The effect was significant when considering the classic CRT, and marginally significant when considering the two items from the extended CRT contained in CRT2. There was no effect of FI or NFC scores.

#### 5.1.4. Regression analysis (base-rate neglect)

Our data forms a perfectly balanced panel with 6 decisions per participant. Table 6 reports random-effects panel regressions on response times, transformed logarithmically. Answers to the alignment versions are significantly slower, also when controlling for personality traits (Model 2). Swiftness is again predictive of the time participants need to work on the base-rate-neglect questions. There is a significant gender effect, suggesting that females are faster in answering the problems.

**Table 6:** **Random-effects regressions on log response times of base-rate-neglect questions**.

**Log(response time)**	**Model 1**	**Model 2**	**Model 3**
Alignment	0.175^***^	0.175^***^	0.126^***^
	(0.049)	(0.049)	(0.027)
Number of words	0.026^***^	0.026^***^	0.025^***^
	(0.002)	(0.002)	(0.002)
Number of letters	−0.005^***^	−0.005^***^	−0.005^***^
	(0.001)	(0.001)	(0.001)
Swiftness		0.174^***^	0.170^***^
		(0.067)	(0.064)
Numeracy		0.041^**^	0.042^**^
		(0.020)	(0.020)
Male		0.156^***^	0.152^***^
		(0.050)	(0.043)
Faith in intuition		0.004	0.004
		(0.019)	(0.019)
Need for cognition		−0.015	−0.014
		(0.025)	(0.026)
Openness to experience		0.012^*^	0.011
		(0.007)	(0.008)
Conscientiousness		−0.004	−0.004
		(0.005)	(0.005)
Extraversion		0.009^***^	0.009^***^
		(0.001)	(0.001)
Agreeableness		−0.008	−0.008
		(0.029)	(0.029)
Neuroticism		0.014	0.015
		(0.016)	(0.017)
Actively open-minded thinking		0.010	0.010
		(0.050)	(0.049)
Error			−0.053
			(0.090)
Error × alignment			0.114
			(0.145)
Observations	942	942	942

The dummy Alignment takes the value 1 for the respective versions of the questions; Conflict is the reference category. Standard errors (in parentheses) are (conservatively) clustered at the level of counterbalance condition (question order).

* p < 0.1,

** p < 0.05,

*** p < 0.01.

Regarding choice data, we ran random-effects probit panel regressions on correct answers to the base-rate-neglect items (Table 7). The variable conflict is significant across all models, indicating an increased likelihood of giving a correct answer to alignment compared to conflict questions. The score obtained in the classic CRT does not affect correct answers across all base-rate-neglect questions (Model 2). Controlling for conflict (Model 3), we obtain the unexpected result that there is a significant negative effect of CRT score on the likelihood of correct answers to alignment questions. Further, the CRT score does not significantly predict a correct answer to the conflict versions (Model 3; *post-hoc* test of the linear combination of Classic CRT plus Conflict × Classic CRT: coefficient 0.010, *SD* = 0.057, *z* = 0.17, *p* = 0.861). In contrast, the score obtained in the two items of CRT2 is a significant positive predictor for correctly answering to the alignment versions of the questions, and also for the conflict versions (Model 3; *post-hoc* test of the linear combination of CRT2 plus Conflict × CRT2: coefficient 0.135, *SD* = 0.059, *z* = 2.30, *p* = 0.022). Results remain stable when including personality traits (Model 4).

**Table 7:** **Random-effects probit regressions on correct answers to base-rate-neglect questions**.

**Correct answer**	**Model 1**	**Model 2**	**Model 3**	**Model 4**
Male	0.032	0.016	0.015	0.014
	(0.134)	(0.129)	(0.130)	(0.153)
Conflict	−1.377^***^	−1.391^***^	−1.639^***^	−1.662^**^
	(0.133)	(0.174)	(0.518)	(0.648)
Classic CRT		−0.037	−0.116^***^	−0.130^*^
		(0.043)	(0.041)	(0.072)
CRT2		0.150^***^	0.174^***^	0.209^***^
		(0.038)	(0.054)	(0.070)
Faith in intuition		0.041	0.050	0.032
		(0.046)	(0.059)	(0.058)
Need for cognition		0.008	−0.005	−0.017
		(0.026)	(0.079)	(0.061)
Numeracy		0.032	0.033	0.030
		(0.036)	(0.037)	(0.034)
Conflict × classic CRT			0.126^**^	0.132^*^
			(0.052)	(0.071)
Conflict × CRT2			−0.039	−0.045
			(0.087)	(0.091)
Conflict × FI			−0.014	−0.017
			(0.030)	(0.030)
Conflict × NFC			0.021	0.025
			(0.087)	(0.087)
Openness to experience				0.071^**^
				(0.036)
Conscientiousness				−0.026
				(0.018)
Extraversion				−0.034^**^
				(0.014)
Agreeableness				0.017
				(0.028)
Neuroticism				0.002
				(0.017)
Actively open-minded thinking				0.052
				(0.066)
Observations	942	942	942	942

The dummy Conflict takes the value 1 for the respective versions of the questions; Alignment is the reference category. Standard errors (in parentheses) are (conservatively) clustered at the level of counterbalance condition (question order).

* p < 0.1,

** p < 0.05,

*** p < 0.01.

#### 5.1.5. Discussion (base-rate neglect)

Response times for questions focusing on base-rate neglect were clearly longer than for the typical CRT questions, with medians above 30 seconds. Such long response times suggest that significant deliberation was involved. The behavioral results (error rates) indicate that in all three pairs, the constructed alignment version was easier than the conflict version. Hence we are confident that the constructed pairs worked as intended. However, the evidence on response times is mixed. For one of the pairs, responses to the alignment question were significantly faster than responses to the conflict version, for another the relation was the opposite, and for the third no significant differences were found. Pooling all the data, a panel regression controlling for swiftness and numeracy indicated a significantly positive effect of alignment on response times. This provides evidence of a tendency for longer response times in the *easier* decisions, which stands in direct contradiction with both Hypothesis H1 and the idea that easier decisions are made faster (Dashiell, 1937; Mosteller and Nogee, 1951).

In Alós-Ferrer and Hügelschäfer (2016), it was already found that responses to different conflict questions used to measure base-rate neglect were affected differently by personality factors. From a conservative point of view, the only conclusion that can be drawn at this point is that, in spite of their apparent similarities at the abstract level, the heavily-framed, context-rich questions might activate quite different processes and process combinations. To fully understand base-rate neglect, and in particular its roots in different decision processes, future research should concentrate on separating framing effects and process conflict or alignment, moving away from the standard questions used in the literature.

The regression analysis allows us to examine the effect of personality differences on both choice data and response times. Scales as FI, NFC, and AOT had no effect in our sample when aggregating across questions. Higher scores in the classic CRT had no effect on error likelihood for the conflict versions of the questions, and surprisingly even increased errors for the alignment versions. In contrast, higher scores in the two items of the extended CRT reduced errors both for conflict and alignment questions. Regarding the Big Five Inventory, Extraversion resulted in longer response times and more errors, and Openness to Experience significantly reduced errors and increased response times.

### 5.2. Conjunction fallacy

#### 5.2.1. Question analysis (conjunction fallacy)

The following four questions refer to the conjunction fallacy. To examine this bias, we employed problems analogous to the classic LINDA question from Tversky and Kahneman (1983).

**(CFC)** Tom is 34 years old. He is intelligent, punctual but unimaginative, and somewhat lifeless. In school, he was strong in mathematics but weak in social studies and humanities. Which of the following statements is more likely to be true?
  ◦ Tom plays in a rock band for a hobby.
  ◦ Tom plays in a rock band for a hobby and is an accountant.
**(CFA)** Klaus is 41 years old, single, introverted, and very intelligent. He majored in physics. As a student, he played pen-and-paper role-playing games, and also participated in several chess tournaments. Which of the following statements is more likely to be true?
  ◦ Klaus DJs on the weekend.
  ◦ Klaus DJs on the weekend or is a university professor.
**(CFN1)** Claire is 30 years old, single, open-minded, and very smart. As a student of literature, she was deeply concerned with issues of discrimination and social justice, and also participated in several demonstrations.Which of the following statements is more likely to be true?
  ◦ Claire is active in the animal-rights movement.
  ◦ Claire is active in the animal-rights movement and works in an international company.
**(CFN2)** Richard is 31 years old, married with no children. A man of high ability and high motivation, he promises to be successful in his field. He is well liked by his colleagues. Which of the following statements is more likely to be true?
  ◦ Richard is an engineer.
  ◦ Richard is an engineer and is active in the civil-rights movement.

The (CFC) item, which is adapted from Tversky and Kahneman (1983) (see also De Neys and Bonnefon, 2013), is analogous to the LINDA problem. Intuition prescribes to select the second option, because the frame seems in line with the stereotype of an accountant more than with that of a rock-band member. This is obviously incorrect, because the simultaneous realization of two disjoint events cannot be more probable than one of the events.

(CFA), (CFN1), and (CFN2) represent different non-conflict versions of the same problem. First, by substituting “and” with “or” in (CFA), the stereotypical answer suggested by the frame becomes logically valid. The change does not affect the mechanism used to correctly answer to the problem, which is still the same as in (CFC). Therefore (CFA) is an alignment counterpart of the conflict item (CFC). Second, the frame of (CFN1) is adapted from the original LINDA problem (Tversky and Kahneman, 1983). By presenting the cue linked to the stereotype (“animal-rights movement”) in both answers, the heuristic which misleads participants in (CFC) cannot directly be applied because it does not have a favored option. Therefore (CFN1) is a neutral counterpart of the conflict item (CFC). Third, (CFN2) is adapted from Kahneman and Tversky (1973). The description of Richard is neutral with respect to the two suggested answers; hence the heuristic process activated in (CFC) is no longer available. Therefore (CFN2) represents another possible neutral counterpart of (CFC).

**Figure 3:**
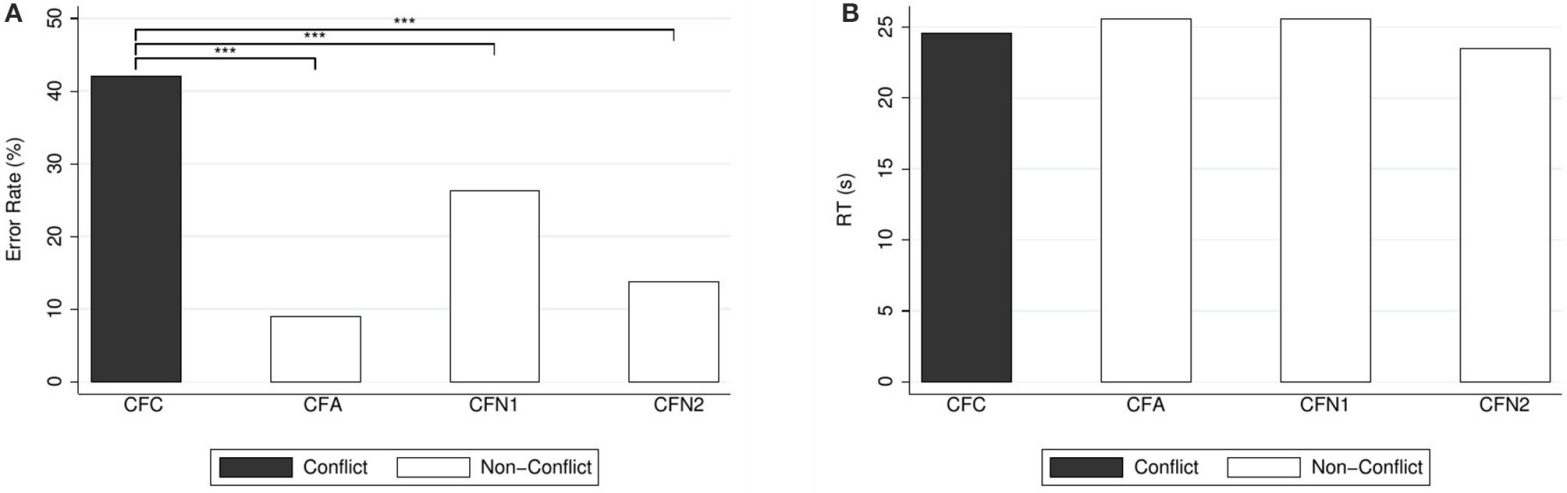
**(A)** Error rates (in %) and **(B)** mean response times (in seconds) for conjunction-fallacy questions. Reported significance refers to McNemar's tests for error rates and to Wilcoxon Signed-Rank tests for response times, as reported in the text. ^***^p < 0.01.

Figure 3 depicts the percentages of errors and correct responses (panel A) and the response times (panel B). We compared the response times in the conflict question to those of the non-conflict variants, but we found no significant differences whatsoever, neither for the alignment question (CFA) (median 24.02 s, mean 25.33 s, *SD* = 7.79, compared to median 23.34 s, mean 24.63 s, *SD* = 9.90 for (CFC); WSR test, *N* = 143, *z* = −1.58, *p* = 0.113) nor for the neutral questions (CFN1) (median 23.61 s, mean 25.32 s, *SD* = 9.32, compared to median 23.24 s, mean 24.67 s, *SD* = 9.99 for (CFC); WSR test, *N* = 144, *z* = −0.64, *p* = 0.524) and (CFN2) (median 22.64 s, mean 23.52 s, *SD* = 8.67 for (CFN2), compared to median 23.14 s, mean 24.58 s, *SD* = 9.93 for (CFC); WSR test, *N* = 145, *z* = 0.78, *p* = 0.437).

We also compared the percentages of errors in the non-conflict questions to those of the conflict question. The proportion of errors in the conflict question was significantly larger than in the alignment question (CFA) [9.09% (13) compared to 42.66% (61) for (CFC); McNemar's test: *N* = 143, $\chi_{\left(1\right)}^{2}=32.91$, *p* < 0.001] and in the neutral questions (CFN1) [27.78% (40), compared to 41.67% (60) for (CFC); McNemar's test: $N=144,\chi_{\left(1\right)}^{2}=7.41,p=0.007$] and (CFN2) [13.79% (20), compared to 42.07% (61) for (CFC); McNemar's test: *N* = 145, $\chi_{\left(1\right)}^{2}=32.96$, *p* < 0.001].

As can be seen from Table 5, the likelihood of a correct answer to the standard conjunction-fallacy problem (CFC) was significantly increased with increasing CRT score when considering the classic 3-item version, but not when considering only the two additional items from Toplak et al. (2014). This result is in line with the findings of Oechssler et al. (2009) and Alós-Ferrer and Hügelschäfer (2016), and also with Liberali et al. (2012), who found a negative correlation between CRT score and number of conjunction fallacies. In contrast, there were no effects of FI and NFC.

#### 5.2.2. Regression analysis (conjunction fallacy)

Our data forms a perfectly balanced panel with 4 decisions per participant. Table 8 reports random-effects panel regressions on response times, transformed logarithmically. The alignment dummy is significantly positive in all three models. The effect of swiftness is as in previous sections. A high score in Need for Cognition is negatively related to response time. Further, the error dummy is significant and positive, meaning that participants making an error in the conflict and neutral versions of the question need more time than participants giving a correct answer. Again, this is a strictly between-subjects comparison which might simply reflect cognitive-capacity correlates.

**Table 8:** **Random-effects regressions on log response times of conjunction-fallacy questions**.

**Log(response time)**	**Model 1**	**Model 2**	**Model 3**
Neutral	−0.056	−0.056	−0.007
	(0.125)	(0.126)	(0.128)
Alignment	0.084^***^	0.084^***^	0.143^***^
	(0.028)	(0.028)	(0.045)
Number of letters	0.005^**^	0.005^**^	0.003^*^
	(0.002)	(0.002)	(0.002)
Swiftness		0.190^***^	0.183^***^
		(0.069)	(0.070)
Numeracy		−0.002	0.004
		(0.022)	(0.023)
Male		−0.012	−0.010
		(0.041)	(0.041)
Faith in intuition		0.005	0.008
		(0.028)	(0.026)
Need for cognition		−0.029^**^	−0.028^**^
		(0.012)	(0.011)
Openness to experience		−0.009	−0.010
		(0.031)	(0.030)
Conscientiousness		0.006	0.007
		(0.014)	(0.014)
Extraversion		0.003	0.003
		(0.011)	(0.011)
Agreeableness		0.013	0.013
		(0.026)	(0.027)
Neuroticism		−0.006	−0.003
		(0.012)	(0.012)
Actively open-minded thinking		−0.005	0.000
		(0.024)	(0.024)
Error			0.156^***^
			(0.048)
Error × alignment			−0.104
			(0.132)
Observations	632	632	632

The dummies Neutral and Alignment take the value 1 for the respective versions of the questions; Conflict is the reference category. Standard errors (in parentheses) are (conservatively) clustered at the level of counterbalance condition (question order).

* p < 0.1,

** p < 0.05,

*** p < 0.01.

To analyze actual choices, we ran random-effects probit panel regressions on correct answers (Table 9). In the basic model, the dummies conflict and neutral are significant and negative, indicating a lower probability of being answered correctly compared to the alignment counterpart. Scoring high in the numeracy scale is associated with an increased probability of giving a correct answer to the questions. Higher scores in the classic CRT are a significant positive predictor for correct answers, in particular for the conflict item (Model 3). This is in agreement with Liberali et al. (2012), who reported a significant negative association between CRT score and committing the conjunction fallacy. In contrast, the number of correct answers to the two items of CRT2 is not predictive (Model 3; *post-hoc* test of the linear combination of CRT2 plus Conflict × CRT2: coefficient −0.136, *SD* = 0.130, *z* = −1.04, *p* = 0.297). Results remain stable when controlling for interindividual heterogeneity by including personality traits (Model 4).

**Table 9:** **Random-effects probit regressions on correct answers to conjunction-fallacy questions**.

**Correct answer**	**Model 1**	**Model 2**	**Model 3**	**Model 4**
Male	0.062	−0.092	−0.088	0.003
	(0.151)	(0.116)	(0.117)	(0.110)
Conflict	−1.173^***^	−1.175^***^	−1.072	−1.128
	(0.226)	(0.226)	(0.788)	(0.775)
Neutral	−0.491^***^	−0.492^***^	−0.489^***^	−0.499^***^
	(0.119)	(0.123)	(0.122)	(0.117)
Classic CRT		0.169^***^	0.089^**^	0.111^*^
		(0.047)	(0.037)	(0.059)
CRT2		−0.063	−0.030	−0.013
		(0.058)	(0.030)	(0.055)
Faith in intuition		0.070	0.076	0.085
		(0.066)	(0.064)	(0.074)
Need for cognition		−0.005	0.012	0.017
		(0.041)	(0.028)	(0.048)
Numeracy		0.081^*^	0.084^*^	0.074^**^
		(0.048)	(0.048)	(0.036)
Conflict × classic CRT			0.250^***^	0.248^***^
			(0.095)	(0.091)
Conflict × CRT2			−0.106	−0.098
			(0.110)	(0.112)
Conflict × FI			−0.014	−0.011
			(0.116)	(0.108)
Conflict × NFC			−0.059	−0.057
			(0.104)	(0.105)
Openness to experience				−0.012
				(0.049)
Conscientiousness				0.020^***^
				(0.006)
Extraversion				−0.001
				(0.026)
Agreeableness				−0.017
				(0.055)
Neuroticism				0.092^***^
				(0.032)
Actively open-minded thinking				0.094
				(0.068)
Observations	632	632	632	632

The dummies Conflict and Neutral take the value 1 for the respective versions of the questions; Alignment is the reference category. Standard errors (in parentheses) are (conservatively) clustered at the level of counterbalance condition (question order).

* p < 0.1,

** p < 0.05,

*** p < 0.01.

#### 5.2.3. Discussion (conjunction fallacy)

Median response times for conjunction-fallacy questions were in the 22–26 s range. Error rates show that the non-conflict versions of the basic (conflict) conjunction-fallacy question were easier. Hence we are confident that the question manipulation worked as intended. However, there were no significant differences in response times. Taking advantage of the panel structure of the data, and controlling for individual differences, the regression revealed a significant positive effect of the alignment question on response times (contrary to Hypothesis H1), but no effect of neutral questions.

One possible explanation for these disappointing results is related to the structure of the questions in detail. By their very nature, these questions seek to consider stereotypes. One alternative presents an event *E*, the other alternative the conjunction of events *E* and *F* (or, in the case of (CFA), their disjunction). In the conflict question (CFC), the frame is stereotypically consistent with *F*, hence “*E* and *F*” becomes an incorrect, intuitive response. In (CFN1) and (CFN2), the intention was to have an event *F* unrelated to the frame, hence shutting down stereotypical thinking. In (CFA), the frame is stereotypically consistent with *F*, but the introduction of a disjunction makes the answer “*E* or *F*” correct.

The process logic operates under the assumption that a stereotype-based, intuitive process will select one answer or the other on the basis of the match between frame and events, and a more deliberative process will operate on the basis of the logic of probability. That the latter is indeed active is evidenced by the sharp drop in the error rate from (CFC) to (CFA), where the disjunction is introduced. However, the characteristics of the stereotypical process might not be fully understood. For instance, in all four questions, there is a basic stereotypical inconsistency between events *E* and *F*. This might activate stereotypical thinking even in the neutral questions (CFN1) and (CFN2), and create a conflict in the alignment question (CFA). In other words, the basic structure of conjunction-fallacy questions might make it difficult to disentangle stereotypical thinking and deliberative processes. Further research should hence try to isolate the actual process involved in stereotypical thinking for this kind of questions.

Personality factors had no effect on response times for conjunction-fallacy questions, with the exception of Need for Cognition, for which higher scores resulted in faster responses. Regarding actual responses, higher scores in the CRT reduced errors (particularly under conflict) as did higher numeracy scores, but FI, NFC, and AOT had no effect. From the Big Five Inventory, only Conscientiousness and Neuroticism had a significant (positive) effect on correct answers, reducing errors.

### 5.3. Ratio bias

The last problem refers to the ratio bias (Kirkpatrick and Epstein, 1992; Denes-Raj and Epstein, 1994), which is the tendency to judge a low-probability event as more likely when it is presented as a ratio of large numbers (e.g., 10 in 100) than as a smaller-numbered ratio (e.g., 1 in 10). For instance, in a study by Denes-Raj and Epstein (1994), a majority of participants preferred an 8-in-100 chance of winning to a 1-in-10 chance of winning.

#### 5.3.1. Question analysis (ratio bias, conflict vs. neutral)

We selected one of the scenarios used by Denes-Raj and Epstein (1994) and complemented it with a non-conflict version as follows.

**(RBC)** There are two urns, a large one containing 100 balls, and a small one containing 10 balls. You must choose one of the urns. A single ball will be extracted at random from the urn you choose, and if the ball is black, you will win. If it is white, you will lose. The small urn contains 1 black ball and 9 white balls. The large urn contains 8 black balls and 92 white balls. Which urn should you choose to maximize the probability of winning?
  ◦ The large urn.
  ◦ The small urn.
**(RBN)** There are two urns, a large one containing 100 balls, and a small one containing 90 balls. You must choose one of the urns. A single ball will be extracted at random from the urn you choose, and if the ball is black, you will win. If it is white, you will lose. The small urn contains 8 black balls and 82 white balls. The large urn contains 8 black balls and 92 white balls. Which urn should you choose to maximize the probability of winning?
  ◦ The large urn.
  ◦ The small urn.

For the (RBC) item, the intuitive but incorrect answer is to select the first option. In (RBN), the number of winning balls in the small urn is changed to make both urns contain the same number of winning balls. The heuristic which led participants in (RBC) to choose the urn with the biggest number of winning balls cannot be applied anymore, but comparing proportions is still possible. Therefore (RBN) is a neutral counterpart of the conflict item (RBC).

**Figure 4:**
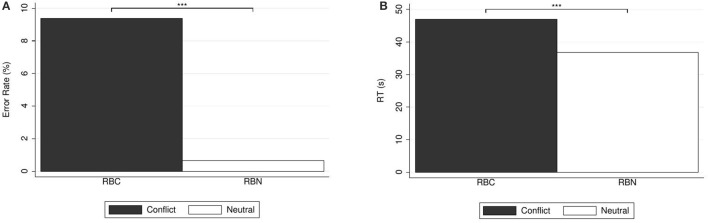
**(A)** Error rates (in %) and **(B)** mean response times (in seconds) for ratio-bias questions. Reported significance refers to McNemar's tests for error rates and to Wilcoxon Signed-Rank tests for response times. ^***^p < 0.01.

Figure 4 depicts the percentages of errors and correct responses (panel A) and the response times (panel B). Answers to the conflict question were significantly slower than the answers to the neutral question (median response time 42.10 s, mean 46.93 s, *SD* = 17.73 in case of conflict; median 35.40 s, mean 36.89 s, *SD* = 12.89 in case of neutral; WSR test, *N* = 149, *z* = 5.36, *p* < 0.001). This is inconsistent with Hypothesis H2 (and also opposite to the results for CRT questions (Q2C) and (Q2N), which did conform to H2).

Regarding error rates, for (RBC) there were 9.40% (14) errors, and 90.60% (135) correct answers. For (RBN) there was only 1 error (0.67%), and all other 148 answers (99.33%) were correct. Of course, the proportion of errors in the conflict question was significantly larger than in the alignment question [McNemar's test, *N* = 149, $\chi_{\left(1\right)}^{2}=11.27$, *p* = 0.001].

As can be seen from Table 5, a higher score in the classic 3-item CRT led to a significantly higher likelihood of answering the (RBC) item correctly. In contrast, none of the scores in CRT2, FI, or NFC affected the likelihood of correct answers. In particular, we fail to reproduce the result of Pacini and Epstein (1999), who reported a more pronounced ratio bias for participants low in NFC.

#### 5.3.2. Discussion (ratio bias)

Error rates were quite low even for the conflict question, but response times were relatively long (medians in the 35–40 s range). The difference in error rates shows that the neutral question worked as intended, with the conflict being removed by shutting down the intuitive process. However, response times were longer for the conflict question, in contradiction with Hypothesis H2. In contrast, the result is compatible with the view that the conflict question induces a struggle between different tendencies which makes the decision more difficult and results in longer deliberation times, analogously to the “closeness to indifference” argument inspired by Dashiell (1937) and Mosteller and Nogee (1951). The dual-process logic under which Hypothesis H2 was derived (which views conflict resolution as a relatively short part of the decision process) might be more appropriate for shorter decisions as those studied for CRT questions, but the “closeness to indifference” view of tradeoffs and conflicts might be more appropriate for long decisions as those related to our ratio-bias questions. This points out to a need for more detailed models of decision processes, especially if they are to encompass relatively long decisions.

## 6. General discussion

Our work provides novel evidence on response times and the multiplicity of decision processes for a category of questions which are extensively used in the decision-making literature. Since responses in experiments in this domain are relatively slow (medians around half a minute or above), our research had an exploratory character.

We selected two kinds of items, those arising from the Cognitive Reflection Test and extensions thereof, and those used to measure decision biases for probability judgments. All such questions create a conflict between an intuitive process and more deliberative thinking, in the terms of dual-process theories. Our strategy of research was to create a non-conflict version for each item, by either making the intuitive impulse correct (resulting in an alignment question), shutting it down (creating a neutral question), or making it dominant (creating a heuristic question).

For CRT items, results were encouraging. The differences in response times are as predicted by dual-process theories, with alignment and heuristic variants leading to faster responses and neutral questions to slower responses than the original, conflict questions. That is, even though response times are relatively long (well above those found in typical experimental workhorses for dual-process theories), evidence is consistent with the involvement of different decision processes and the diagnosticity of their interaction (conflict or not).

For decision-bias items, results were sobering. Results on conflict vs. alignment for base-rate neglect questions were inconclusive on the aggregate in spite of significant effects for some individual items. In our opinion, this points out that the heavily-framed questions employed in this area are not stylized enough to properly identify the involved processes, and further efforts are needed in order to disentangle framing and the effects of conflict or alignment among decision processes. For the conjunction fallacy, response-time differences were generally not significant, even though the manipulations worked as intended in terms of error rates. In view of the structure of the items, we tentatively conclude that stereotypical thinking cannot be properly isolated with the standard frames used to study the conjunction fallacy, and recommend further research to move away from this basic structure. For the ratio-bias item, where response times are particularly long, we obtained a clear result showing that a neutral version of the original, conflict question results in lower error rates and shorter response times. This is compatible with the view that, in this case, process conflict reflects a stronger behavioral struggle resulting in longer deliberation (following a classic “closeness to indifference” argument).

### 6.1. Response times and underlying assumptions

It is worth discussing possible explanations for the differences in results between the CRT items and the items on decision biases. Two avenues are apparent, one procedural and one conceptual.

The procedural avenue concerns the fact that our implementation of the decision-bias items involved binary choices, while the CRT items were open-ended [with the exception of (Q4C) and (Q4A)]. The reason is that, for the CRT items, the exactly correct answer is still reasonably easy to arrive at, and the alternative, intuitive process provides a specific answer. Hence the open-answer format is natural. In contrast, for the base-rate questions the postulated processes do not deliver precise answers. Correct answers are the result of complex, precise calculations while “intuitive” tendencies have a directional nature (high or low probability estimate). Hence, we presented those items with binary-choice answers (larger or smaller than 50%). However, it is unlikely that this procedural difference is determinant for the difference in results. First, we did not compare response times of different answers for a fixed question, but rather the response times for different *questions*. Whatever answers the different processes led to, differences among types of questions should subsist. Second, for the items related to the conjunction fallacy and the ratio bias, the binary-choice format is indeed natural, because the correct response is easy to arrive at, and the alternative intuitive processes do provide a clear response. However, it remains at least conceivable that for the base-rate-bias item pairs, the presentation of binary-choice answers lowered participant involvement (since there was no need to arrive at an exact numerical estimate), hence activating decision processes different from those postulated in the analysis. This, however, would have no bearing on the conjunction-fallacy and ratio-bias items.

The conceptual avenue arises from our discussion in Section 2.4. Our hypotheses are always derived from the confluence of two effects. First, the time required for conflict detection and resolution should be smaller if there is no actual conflict. Second, the kind of question should affect the percentage of intuitive (hence faster) decisions. Evidence on conflict detection and resolution, however, indicates that the required time might be relatively short. Specifically, EEG research shows that conflict detection and resolution are probably associated with activity in the anterior cingulate cortex occurring as early as 200 milliseconds (see, e.g., Nieuwenhuis et al., 2003; Coderre et al., 2011; Achtziger et al., 2014). Although this evidence has been gathered for paradigms with simple stimuli (suitable for EEG analysis), it can be speculated that even for paradigms with longer response times as the ones considered here, the time necessary for conflict detection and resolution in the sense of dual-process theories is relatively short (if at all relevant), implying that response-time effects should be driven by the second phenomenon described above, namely the shift in likelihood from one type of process to the other.

If, for the sake of the argument, we accept this preliminary hypothesis, we can reexamine our results. Evidence from our CRT item pairs is compatible with the postulate that, relative to conflict items, the balance is shifted toward automatic processes in case of alignment, and obviously toward deliberative processes for neutral items. Since the former are faster than the latter, this observation suffices to explain our data. It is also compatible with the fact that smaller error rates are observed in both cases, since in case of alignment intuitive processes also deliver the correct answer.

For our decision-bias items, as shown in the analysis sections above, it remains true that error rates for alignment and neutral items are lower than for the corresponding conflict items (the comparison was significant in every single case). Hence, based on the error-rate evidence, we have no reason to doubt that in every item pair, the process shift occurred as postulated. However, response-time evidence appears inconsistent. At this point of the argument, the original response-time predictions rest on a single assumption, namely that the expected response times of the deliberative processes for these items are indeed always larger than the expected response times of the corresponding intuitive processes (*T*_*U*_ > *T*_*H*_). Given the simple nature of the processes involved in the CRT questions, there is little reason to question this assumption in that setting. For decision-bias items, however, it has been argued that many of the involved heuristics might not be fast shortcuts, but rather “cognitive heuristics” including multi-step operations (even if they are sometimes called “fast and frugal,” Gigerenzer and Goldstein, 1996). If this is the case for the intuitive processes involved in decision biases of the type examined here, then the assumption of a significant difference in expected response times among processes for this particular case might not be justified. Our data is consistent with this interpretation, but further evidence is needed.

### 6.2. Long response times and types of decisions

At this point, we can conclude that the scope of response times has a definite influence on their interpretation. For short response times it is comparatively easier to identify the involved decision processes and simple dual-process models deliver instructive predictions. For longer decisions, the exact length thereof might reflect moderators of deliberation, and predictions should be more modest at this point. Clearly, there is a need for improved models of deliberation and the associated process data.

It should be kept in mind that we have concentrated on decisions from inference where an objectively correct decision can be identified in advance and natural hypotheses on the nature of the involved processes are available. This is in stark contrast to preferential-choice settings, where the nature of the involved processes is open to discussion. For instance, Cappelen et al. (2015) examined response times in the dictator game and argued that choosing a fair allocation of resources among two people (as opposed to keeping most of a given resource for oneself) might be more intuitive, because the average response times were shorter (but still quite long). As observed by Myrseth and Wollbrant (2016), this might amount to a reverse-inference fallacy, especially since the conclusion is not based on a theoretical model, but rather operates as if there was a one-to-one correspondence between processes and choices (see Alós-Ferrer, 2016 for a discussion of this point). Further, preferential choice presents an added difficulty. We have concentrated our analysis on response-time differences *across questions*, which enable paired comparisons of data. This is important because there exists a large response-time heterogeneity across individuals, which becomes exacerbated for long response times as the ones we study. In studies of preferential choice as Cappelen et al. (2015), there is exactly one observation per individual, and the population of subjects is partitioned according to the response. Hence, individual heterogeneity is harder to control for. This is why our analysis focused on paired-observations tests, moving to regressions only to clarify the possible effect of additional individual correlates.

### 6.3. Personality measures

Our analysis also points out the necessity of further research on the influence of personality traits on decision-making biases. In spite of some clear general trends, evidence is still mixed. We found that higher scores in the CRT resulted in significantly more correct responses for both the conjunction fallacy and the ratio bias. However, we did not find a clear predictive effect of higher scores in the CRT on correct responses for base-rate-neglect questions. Faith in Intuition, Need for Cognition, and Actively Open-Minded Thinking were generally non-predictive for correct responses in our sample. However, we used the short REI-10 version with 5 items per subscale, while Alós-Ferrer and Hügelschäfer (2016) used a 15-item version.

Regarding the Big Five Inventory, we confirmed the typical correlations with other personality traits found in the literature. We included them as controls in regressions on both choices and response times for the base-rate-neglect and conjunction-fallacy items. We found significant effects, but none of the five personality traits showed a consistent effect for base-rate neglect and the conjunction fallacy. For instance, Extraversion resulted in more base-rate-neglect errors but had no effect on the conjunction-fallacy items. This is especially interesting, because this personality trait has been related to a more sensitive midbrain dopaminergic reward system, leading to difficulties in regulating impulsiveness (Depue and Collins, 1999; Cohen et al., 2005).

We conclude that the effects of personality measures often appear to be bias-specific, and apparently related constructs, which are supposed to measure related traits, often have different effects. The CRT is predictive for different decision biases, but the scale is becoming generally known and, contrary to self-report questionnaires, cannot be reliably measured repeatedly. Subscales from the Rational-Experiential Inventory have a predictive value (recall Alós-Ferrer and Hügelschäfer, 2012, 2016), but the effects appear to be small in general. Personality traits from the Big Five Inventory often have significant effects, but those are generally inconsistent across biases. Larger datasets, allowing for the study of multiple interactions, might contribute to obtain a more clear picture.

## Author contributions

All authors contributed equally to this work. The listing of authors is alphabetical.

### Conflict of interest statement

The authors declare that the research was conducted in the absence of any commercial or financial relationships that could be construed as a potential conflict of interest.

## Footnotes

^1^The counterbalance condition did not significantly affect participants' response times or responses to any of the questions.
